# Recent advances in the treatment of pancreatic cancer

**DOI:** 10.12688/f1000research.21981.1

**Published:** 2020-02-21

**Authors:** Marc T Roth, Dana B Cardin, Jordan D Berlin

**Affiliations:** 1 Department of Hematology and Oncology, Vanderbilt-Ingram Cancer Center, Nashville, TN, USA

**Keywords:** pancreatic cancer, review

## Abstract

Pancreatic ductal adenocarcinoma is one of the deadliest solid tumor malignancies and is projected to become a leading cause of cancer-related death in coming years. Improving quality of life and survival amongst these patients will require new ideas and novel therapies in a multidisciplinary approach. This review will cover the most recent advances in the comprehensive treatment of pancreatic cancer and place them within a historical context when necessary. Treatment of all disease stages will be discussed, but the focus is on systemic therapy as novel drugs and new treatment combinations enter the clinic. This will include more aggressive chemotherapy in earlier disease stages, approved uses for immunotherapy, and targetable mutations. In addition, negative trials of importance and controversial topics will be noted.

## Introduction

Pancreatic cancer has one of the highest mortalities of all malignancies. It is estimated that 56,770 diagnoses were made and 45,750 deaths occurred in 2019 in the US ^1^. One analysis projects that pancreatic cancer will surpass breast, prostate, and colorectal cancers as the leading cause of cancer-related death in the US by the year 2030 ^2^. These estimates highlight the urgent need for new and innovative treatment options for patients with this deadly disease. Indeed, numerous efforts are under way to alter the disease course in order to keep pace with the improved outcomes seen in other malignancies. The aims of this review are to highlight these advancements and to revisit the historical basis for current treatment options in pancreatic ductal adenocarcinoma (PDAC).

## Chemotherapy

Chemotherapy remains a cornerstone of treatment for PDAC in all stages of disease. Recent data and US Food and Drug Administration (FDA) approvals are divided into three major treatment categories: adjuvant, unresectable/metastatic, and neoadjuvant/induction treatments.

### Adjuvant setting

The role of adjuvant chemotherapy in PDAC was established in multiple trials, including ESPAC-1, CONKO-001, and ESPAC-3 ^3, 4^. ESPAC-4 compared the combination of gemcitabine/capecitabine with gemcitabine monotherapy in the adjuvant setting ^5^. This study, published in 2017, found that the rates of median overall survival (mOS) were 28.0 months in the combination arm and 25.5 months in the monotherapy arm (hazard ratio [HR] 0.82, 95% confidence interval [CI] 0.68–0.98; *P* = 0.032). GI PRODIGE 24, published in 2018, compared adjuvant modified FOLFIRINOX—infusion 5-fluorouracil (5-FU), folinic acid, irinotecan, and oxaliplatin—with gemcitabine ^6^. The term “modified” refers to the reduction in irinotecan dosing from 180 to 150 mg/m ^2^ and the omission of the 5-FU bolus. These changes were made after concerns with the triplet regimen arose during a protocol-specified safety analysis. The mOS rates were an unprecedented 54.4 months for modified FOLFIRINOX and 35.0 months in the gemcitabine arm. It is important to note that those eligible for modified FOLFIRINOX included patients who had fully recovered from surgery and had a performance status of 0 to 1 and adequate hematologic and hepatologic function, making it important to assess the fitness of adjuvant therapy candidates. Gemcitabine/capecitabine and modified FOLFIRINOX are the current standards of care in the adjuvant setting for appropriate patients with resected PDAC.

One notable negative study (APACT, ClinicalTrials.gov Identifier: NCT01964430) compared gemcitabine and *nab*-paclitaxel (Abraxane ^®^, Celgene Corporation, Summit, NJ, USA) (G/A) with gemcitabine monotherapy. The trial did not meet its primary endpoint of improvement in disease-free survival when compared with gemcitabine alone ^7^.

### Unresectable/metastatic disease

Many of the cytotoxic regimens discussed previously were first studied in patients with metastatic disease. The ACCORD trial, published in 2011, compared FOLFIRINOX with gemcitabine in metastatic PDAC (mPDAC), resulting in mOS rates of 11.1 months in the triplet arm and 6.8 months with monotherapy (HR 0.57, 95% CI 0.45–0.73; *P* <0.001) ^8^ and leading to a new standard of care for patients with advanced disease with a good performance status. The MPACT trial, published in 2013, evaluated G/A in mPDAC, finding improved mOS rates of 8.5 months in the combination arm and 6.7 months with gemcitabine alone (HR 0.72, 95% CI 0.617–0.835; *P* <0.001) ^9^, again adding a new regimen to the standard-of-care options for patients with advanced disease. In 2015, the NAPOLI-1 trial compared 5-FU/nanoliposomal irinotecan (nal-IRI) with either drug as monotherapy in metastatic PDAC previously treated with gemcitabine ^10^. In this phase III randomized controlled trial (RCT), 417 patients were enrolled. The mOS rates were 6.1 months in the combination arm and 4.2 months in the 5-FU arm (HR 0.67, 95% CI 0.49–0.92; *P* = 0.012). Of note, mOS in the nal-IRI arm was 4.9 months. This regimen is currently listed as category 1 in the second-line setting for metastatic disease by the National Comprehensive Cancer Network (NCCN) ^11^ and is important as a rare randomized trial in this setting, especially one demonstrating the potential benefit of salvage chemotherapy in a difficult-to-treat patient population. Most recently, the combination of gemcitabine, *nab*-paclitaxel, and cisplatin was tested in a phase Ib/II trial in metastatic PDAC with a primary endpoint of complete response rate (CRR) (25% needed for significance) ^12^. Although this benchmark was not met (CRR 8%), the triplet did yield an overall response rate (ORR) of 71% and mOS of 16.4 months (95% CI 10.2–25.3). The triplet continues to be tested in the neoadjuvant setting and in advanced biliary cancer.

Although cytotoxic chemotherapy is responsible for the largest survival advantages seen in the metastatic setting, novel agents are actively being investigated. Pegvorhyaluronidase alfa (PEGPH20), which degrades hyaluronan in the extracellular matrix, was the subject of much interest in recent years until development was ceased in late 2019 by Halozyme Therapeutics. Although phase II data showed an improvement in progression-free survival (PFS) when combined with G/A ^13^, the triplet failed to meet its primary endpoint of OS in a phase III placebo-controlled RCT (11.2 versus 11.5 months, HR 1.00; *P* = 0.9692) ^14^. AVENGER 500 (ClinicalTrials.gov Identifier: NCT03504423) is a phase III RCT investigating modified FOLFIRINOX with or without CPI-613 ^15^, a tricarboxylic acid cycle inhibitor. Accrual should be complete by mid-2020, and results are highly anticipated after showing promise in the phase I setting (ORR 61%, CRR 17%) ^16^.

### Neoadjuvant and induction treatment

Since establishing the efficacy of chemotherapy in the adjuvant setting and advanced disease, its movement into the pre-operative space has been a logical progression. The rationale behind this includes potentially increasing the R0 resection rate, administering more therapy prior to surgery when it is better tolerated, and providing time to evaluate the biology of a patient’s individual disease. In addition, as criteria for resection change and procedures become more extensive, conversion or downstaging therapy with chemotherapy in the borderline resectable pancreatic cancer/locally advanced pancreatic cancer (BRPC/LAPC) space is becoming commonplace. Multiple institutional and small multi-center trials have been conducted to determine the optimal chemotherapy regimen or utility of radiation (or both) in this space, although currently there is no established standard of care in this arena. The spectrum of radiographic staging and sequence of treatment in these trials can make extrapolation into common clinical practice difficult.

Several ongoing trials are investigating pre-operative chemotherapy for upfront resectable PDAC. One such study is the phase II/III Prep-02/JSAP-05 (UMIN000009634), which compared gemcitabine/S1 followed by resection with upfront surgery, and OS was its primary endpoint ^17^. The mOS rates were 36.7 months in the chemotherapy arm and 26.6 months with upfront surgery alone (HR 0.72, 95% CI 0.55–0.94; *P* = 0.015). Resection rate, R0 rate, and morbidity were similar between the two arms. Further studies should be performed in this space to inform generalization to the broader PDAC population.

Current practice for many institutions in the US for fit patients with borderline resectable PDAC is neoadjuvant modified FOLFIRINOX with consideration of radiation therapy (RT) followed by resection if appropriate. This stems from the phase I single-arm ALLIANCE trial A021101 (published in 2016), in which patients who received the above regimen achieved an mOS of 21.7 months (95% CI 15.7–not reached) and a 68% resection rate (95% CI 49–88%) ^18^. The confirmatory trial is ongoing (A021501, discussed below). NEOLAP (ClinicalTrials.gov Identifier: NCT02125136) is the first randomized trial comparing FOLFIRINOX and G/A in LAPC. In that study, patients were given two cycles of G/A induction and then randomly assigned to either two additional cycles of G/A or four cycles of FOLFIRINOX. Final results presented at the European Society for Medical Oncology annual congress in 2019 were notable for conversion rates of 30.6% for G/A and 45.0% for FOLFIRINOX, although this difference was not considered statistically significant (odds ratio 0.54, 95% CI 0.26–1.13; *P* = 0.135) ^19^. The highly anticipated phase II SWOG 1505 (ClinicalTrials.gov Identifier: NCT02562716) trial moves the comparison of G/A with FFX entirely into the upfront resectable setting with a “pick the winner” design using 2-year OS as the primary endpoint ^20^. Other ongoing studies will add to the growing body of evidence in this arena. NEONAX (ClinicalTrials.gov Identifier: NCT02047513), a phase II randomized study, has recently completed accrual. That trial compares two pre-operative and four post-operative cycles of G/A with six cycles of adjuvant G/A.

## Radiation

Evidence regarding the role of RT in pancreatic cancer has been conflicting, although its use has been of great interest. Cross-trial comparisons are even more difficult with RT given that the dose and mode of radiation delivery have changed over the years. LAP07 was one of the largest randomized trials to evaluate the role of gemcitabine and erlotinib with or without chemoradiation in LAPC. The trial was stopped early for futility as no significant survival difference was noted between the chemoradiotherapy- and chemotherapy-alone arms ^21^. Few definitive practice-changing trials have been conducted recently, but two studies hoping to clarify the role of RT in this space are ongoing. The ALLIANCE trial A021501 (ClinicalTrials.gov Identifier: NCT02839343) is comparing neoadjuvant FOLFIRINOX followed by stereotactic body RT (SBRT) with FOLFIRINOX alone followed by resection (if possible) in borderline resectable PDAC ^22^. Interestingly, the radiation arm has since closed because of futility. Results of the interim analysis have yet to be reported but will be an intriguing addition to an ongoing discussion. The PREOPANC-1 (NTR3709) trial is a phase III RCT comparing upfront resection to gemcitabine-based chemoradiation followed by resection, and both arms are receiving adjuvant gemcitabine. Though preliminary, data suggest an improved OS (17.1 versus 13.5 months, HR 0.71; *P* = 0.047) and R0 resection rate (65% versus 31%; *P* <0.001) with pre-operative chemoradiation compared with chemotherapy alone ^23^.

In 2019, the American Society for Radiation Oncology released new guidelines for the treatment of pancreatic cancer ^24^. These recommended the consideration of conventionally fractionated RT or SBRT in high-risk adjuvant settings, as neoadjuvant downstaging for BRPC/LAPC, and for LAPC as part of definitive treatment, all admittedly with low to moderate quality of evidence. It should be noted that in contrast to other data presented regarding efficacy of chemotherapy in PDAC, the use of radiation is not founded on solid evidence as of yet.

## Immunotherapy

PDAC has been largely excluded from the recent success stories in the field of immunotherapy (IO) relative to many other malignancies. Single- and double-agent IO, as well as combinations with cytotoxic chemotherapy and radiation among others, have been studied but have not shown meaningful clinical benefit ^25^. There are multiple hypotheses for the underlying mechanism of resistance to IO in PDAC. These include a lack of effector cells in the tumor microenvironment combined with an immunosuppressive infiltrate, dense stroma impairing migration of effector cells, and immune checkpoint signaling ^26^. Many studies are under way to discover the key to overcoming these barriers. It is important to highlight that IO, though used in many other malignancies, is not approved for the treatment of PDAC outside of clinical trials.

Currently, the only exception to this is in PDAC with microsatellite instability (MSI). In May 2017, the FDA granted its first tissue/site-agnostic approval to pembrolizumab for the treatment of MSI-high or deficient mismatch repair (dMMR) solid tumors on the basis of data from five single-arm trials. One enrolled 86 patients, including eight patients with PDAC ^27^. That study had co-primary endpoints of immune-related PFS and ORR. In the intention-to-treat population, the ORR was 53% (95% CI 42–64%) and PFS rates at 1 and 2 years were 64% and 53%, respectively. Among patients with PDAC, the ORR was 62%, and two patients (25%) achieved a complete response. Updated results from the KEYNOTE-158 study are less promising. Among 22 patients with PDAC, the ORR was 18.2%, the median PFS (mPFS) was 2.1 months, and the mOS was 4.0 months ^28^.

## Targeted therapy

Recent large-scale molecular profiling efforts have attempted to uncover genomic subtype prevalence and corresponding therapeutic relevance in PDAC. One such effort found that 14% of evaluated patients harbored mutations (either germline or somatic) in *BRCA1*, *BRCA2*, and *PALB2* ^29^. Similarly, initial results from the Know Your Tumor initiative showed that 27% of evaluated patients had highly actionable mutations, which when paired with matched therapy resulted in a longer mPFS than that of patients without matched therapy ^30^. A number of other genes are frequently mutated in PDAC ( *KRAS*, *TP53*, *CDKN2A*, *SMAD4*, *MLL3*, *TGFBR2*, *ARID1A*, and *SF3B1*) but as of yet do not have therapeutic indications though may provide detail on prognosis ^31^. Neurotrophic receptor tyrosine kinase ( *NTRK*) gene fusions have been found in a small fraction of PDAC, and some estimates are as high as 6% ^32^. Current options for targeted treatment in PDAC are discussed below.

### NTRK fusions

There are two new treatment options for patients found to have fusion proteins in the *NTRK* gene detected by next-generation sequencing or fluorescence *in situ* hybridization. Larotrectinib ^33^ was granted accelerated approval in November 2018 on a tissue-agnostic basis for unresectable or metastatic solid tumors harboring the gene fusion. This approval was based on data from three single-arm clinical trials: LOXO-TRK-14001, SCOUT, and NAVIGATE ^34^. In total, 55 patients (children and adults) were enrolled between the trials. At the time of assessment, the primary endpoint of ORR was 75% (95% CI 67–90%) and the mPFS had not been reached after a median follow-up duration of 9.9 months. It should be noted that only one patient with pancreatic cancer was included, although that person was among the responders. The drug was generally well tolerated; grade 3 and higher adverse events (AEs) occurred in less than 5% of patients. The most common side effects were aspartate aminotransferase/alanine aminotransferase (AST/ALT) abnormalities, fatigue, and vomiting. **

Entrectinib ^35^ was granted accelerated approval for the same indication in August 2019 on the basis of three additional single-arm trials: ALKA-372-001, STARTRK-1, and STARTRK-2 ^36^. Between the two phase I studies, 60 patients harboring a gene rearrangement in *NTRK*, *ROS1*, or *ALK* were enrolled. Given their early phase, only preliminary efficacy data are available, but in the three evaluable patients with *NTRK* fusions, the ORR was 100% (95% CI 44–100%), although none had PDAC. The phase II study (STARTRK-2) is under way. The most common side effects were fatigue, dysgeusia, and parasthesias.

### Homologous recombination deficiency

Mutations in homologous recombination deficiency (HRD) genes (including *BRCA1/2*) have been correlated with defective DNA repair ^29^. Given the inherent genomic instability in this setting, various interventions to exploit this and induce apoptosis have been studied in PDAC. Patients with germline mutations in *BRCA1/2* appear to have an increased sensitivity to platinum agents ^37^, and the NCCN recommends treatment of this subpopulation with gemcitabine plus cisplatin in locally advanced and metastatic disease ^11^.

A more recent mechanism to leverage this pathway involves poly(adenosine disphosphate-ribose) polymerase (PARP) inhibition. The POLO trial was a phase III RCT evaluating olaparib (a PARP inhibitor) maintenance versus placebo in patients with metastatic PDAC and germline *BRCA1/2* mutations whose disease did not progress after first-line platinum-based chemotherapy ^38^. The primary endpoint was PFS. mPFS rates were 7.4 months in the treatment arm and 3.8 in the control arm (HR 0.53, 95% CI 0.35–0.82; *P* = 0.004) but this did not equate to an OS advantage during the interim analysis (mOS 18.9 versus 18.1 months, respectively; HR for death, 0.91; 95% CI 0.56–1.46; *P* = 0.68). Rates of grade 3 and higher AEs were 40% in the treatment group and 23% in the placebo group, and the most common AEs were fatigue, nausea, diarrhea, and abdominal pain. In December 2019, the FDA approved olaparib for use in those who had germline *BRCA1/*2 mutations, a good performance status, and no disease progression after at least 4 to 6 months of chemotherapy. Further studies are ongoing with novel drug combinations targeting a wider range of DNA damage repair pathways in PDAC.

## Summary

Patients with pancreatic cancer today have increased options for treatment relatively speaking, but survival for this disease is dismal and falling behind in a rapidly advancing field. Clinical trial design must evolve to address this need, and trial participation should be considered whenever possible. Established questions such as the role of radiation in pancreatic cancer and the precise populations that will benefit remain unanswered. New issues regarding resistance to IO and the role of targeted therapy have also been presented. Progress is being made, however, from pharmaceutical innovation to an increasing number of studies including the elderly and those with a poor performance status and refractory disease. With such efforts, there is hope that more effective treatment strategies are within reach.
